# New Insights into the Inhibition of Hesperetin on Polyphenol Oxidase: Inhibitory Kinetics, Binding Characteristics, Conformational Change and Computational Simulation

**DOI:** 10.3390/foods12040905

**Published:** 2023-02-20

**Authors:** Xinyue Hong, Xiaoqiao Luo, Langhong Wang, Deming Gong, Guowen Zhang

**Affiliations:** 1 State Key Laboratory of Food Science and Technology, Nanchang University, Nanchang 330047, China; 2 School of Food Science and Engineering, Foshan University, Foshan 528225, China

**Keywords:** hesperetin, polyphenol oxidase, inhibition mechanism, conformational change, computational simulation

## Abstract

The inhibitory activity of hesperetin on polyphenol oxidase (PPO) and their interaction characteristics were investigated using multiple spectroscopic methods and computational simulation. Hesperetin, a mixed inhibitor, reversibly inhibited PPO activity, and its half-maximum inhibitory concentration (IC_50_) values on monophenolase and diphenolase were 80.8 ± 1.4 μM and 776.0 ± 15.5 μM, respectively. Multivariate curve resolution–alternate least squares (MCR–ALS) analysis suggested PPO interacted with hesperetin and formed PPO–hesperetin complex. Hesperetin statically quenched PPO’s endogenous fluorescence, and hydrophobic interactions mainly drove their binding. Hesperetin affected the polarity of the microenvironment around the Trp residues in PPO, but had no effect on that around Tyr residues. Circular dichroism (CD) results showed that hesperetin increased α-helix content and decreased β-fold and random coil contents, thus tightening PPO’s structure. Molecular docking showed that hesperetin entered the hydrophobic cavity of PPO, bound near the dinuclear copper active center, interacted with Val283, Phe264, His85, Asn260, Val248, and His263 via hydrophobic interactions, formed hydrogen bonds with Met280, His89, and His259 residues and also interacted with Phe292, His61, Phe90, Glu256, His244, Asn260, Phe264, and Gly281 via van der Waals forces. The molecular dynamics simulation results also demonstrated that the addition of hesperetin reduced the stability and hydrophobicity of PPO and increased PPO’s structural denseness. Thus, the inhibition of hesperetin on PPO may be because hesperetin bound near the active center of PPO, interacted with the surrounding residues, occupied the binding site for substrate, and induced the changes in PPO’s secondary structure, thus inhibiting the catalytic activity of PPO. This study may provide novel views for the inhibition of hesperetin on PPO and theoretical guidance for developing flavonoids as new and efficient PPO inhibitors.

## 1. Introduction

Polyphenol oxidase (PPO), also called tyrosinase, can cause the browning of vegetables, fruits and shrimps in the food industry [1]. As a rate-limiting enzyme related to enzymatic browning reaction, it catalyzes monophenols to diphenols and further oxidizes diphenols to o-quinone, finally transforming to melanin [2]. The conversion of phenols to melanin not only decreases the nutrition of foods and produces some harmful substances, but also causes food to show an unacceptable color and lose commodity value [3]. Thus, it is worthwhile to inhibit the activity of PPO to avoid food waste.

The traditional ways to reduce the activity of PPO in food can be divided into physical and chemical methods. Physical methods usually include heating treatment, high-pressure treatment and UV treatment, and chemical methods usually involve adding acidifiers (citric acid, ascorbic acid, etc.), or sulfur dioxide and its sulfites [4]. However, when the above methods inactivate PPO, they may also destroy the nutrients in food. For instance, Zhang et al. [5] reported that heating treatment caused the degradation of anthocyanins in eggplant peel and the reduction of its antioxidant activity. The amount of an acidifier added is critical in practice: too little, and the inhibitory effect is not enough to avoid the browning of food; too much, and the severe acidification of food may affect its flavor, and excess ascorbic acid can even aggravate browning during storage [6]. Based on these, new approaches are increasingly being used to inhibit the activity of PPO and much attention has been paid to natural extracts for applications in the food industry. The reason may be that the substances from natural sources not only have the capacity to inhibit PPO in food but can also provide nutritional supplements [7].

Many researchers have found that the natural substances from plants, such as herbs, or foods, such as vegetables and fruits, possess an effective inhibitory ability on PPO, which could avoid food browning. For example, Siew et al. [8] reported that cinnamon bark inhibited the PPO from Musa acuminata ‘*Mas*’ peel due to its hydrophobic bioactive constituents. Yu et al. [9] found that *Rosa roxburghii* juice effectively prevented apple juice from browning and that its anti-browning ability was related to its content of ascorbic acid and phenolic compounds. Among them, many flavonoids, such as quercetin [10], dihydromyricetin [11], apigenin [12] and naringenin [13] inhibited PPO activity in different manners (competitive, mixed or non-competitive types). Their inhibitory effects are often explained by flavonoids chelated with copper ions or induced conformational changes of PPO [10].

Hesperetin as a dietary flavonoid widely exists in rutaceae citrus fruits, such as lemons and oranges, and has multiple biological activities, including anti-inflammation, anti-oxidation [14], anti-cancer [15] and anti-Alzheimer’s disease [16]. Si et al. [17] investigated the inhibition mechanism of hesperetin on PPO using inhibition kinetics and molecular docking. They reported that hesperetin was a competitive PPO inhibitor and attributed the inhibition of hesperetin against PPO to the fact that two Cu^2+^ were located at the active center of the enzyme and could be reversibly chelated by hesperetin. However, to our knowledge, there were no detailed studies on the conformation of PPO influenced by hesperetin, which may also be one of the crucial reasons for the inhibition of hesperetin on PPO. Therefore, the inhibitory mechanisms of hesperetin against PPO were further investigated, particularly the binding characteristics of hesperetin and PPO, and how hesperetin affected the conformation of PPO was explored using multiple spectroscopic methods combined with computational simulation. This study may provide novel insights into the inhibition mechanisms of hesperetin against PPO and theoretical guidance for developing flavonoids as new and efficient PPO inhibitors.

## 2. Materials and Methods

### 2.1. Materials

PPO (from mushroom, EC 1.14.18.1, 128 kDa) was obtained from Worthington Biochemical Co. (Lakewood, CO, USA). Hesperetin (purity ≥ 98%), L-tyrosine, and L-dopa were obtained from Aladdin Biochemical Technology Co. (Shanghai, China). Kojic acid (KA, purity > 99%) was acquired from Oddfoni Biological Technology Co. (Nanjing, China). Other chemicals used were of analytical grade.

### 2.2. Methods

#### 2.2.1. PPO Activity Assay

To determine the monophenolase inhibitory activity of hesperetin, PPO (0.2 μΜ) was mixed with various concentrations of hesperetin (0, 25, 50, 100, 150, 200, 250 and 500 μM), and the mixtures were incubated at 30 °C. After 1 h, L-tyrosine (0.5 mΜ, substrate) was added to the mixtures, and the detection began. The absorbance values were recorded at 475 nm for 30 min (121 times per 15 s) using a microplate reader (Varioskan LUX, Thermo Scientific, Waltham, MA, USA) to determine the oxidation of L-tyrosine [18].

Based on a previous method [19], the diphenolase inhibitory activity of hesperetin was determined spectrophotometrically by measuring the rate of L-dopa converted to dopaquinone. A series of assay solutions containing 20 μL PPO (20.0 μΜ) and various amounts of hesperetin (20.0 mM) were incubated at 30 °C. After 1 h, 200 μL L-dopa (5.0 mM, substrate) was added to start the action. The absorbance values were continuously monitored at 475 nm for 200 s (41 times per 5 s) by a UV–vis spectrophotometer (UV–2450, Shimadzu, Tokyo, Japan). Kojic acid was used as a positive control [20]. The inhibitory capacity of samples with different concentrations was expressed through relative PPO activity. The PPO activity without an inhibitor was defined as 100%, and the relative activity of PPO suppressed by different samples; relative activity (%) = *K*/*K*_0_ × 100%, where *K* and *K*_0_ represent the slopes of the absorbance, changed over time with and without an inhibitor, respectively [21].

#### 2.2.2. Kinetic Analysis of Inhibitory Type

The inhibitory type of hesperetin against PPO could be evaluated using Lineweaver–Burk plots, and the mixed inhibition was described through Equation (1) [22]:

(1) $$\frac{1}{v}=\frac{K_{m}}{V_{\max}^{}}(1+\frac{\left[I\right]}{K_{i}^{}})\frac{1}{\left[S\right]}+\frac{1}{V_{\max}^{}}(1+\frac{\left[I\right]}{\alpha K_{i}^{}})$$

where *v* and *V*_max_ represent enzyme reaction velocity and its maximum value, respectively. [I], [S] and *K_m_* are the concentrations of hesperetin and L-dopa, and the Michaelis–Menten constant, respectively. *α* denotes the apparent coefficient and *K_i_* represents dissociation constant of hesperetin binding with free PPO, which can be calculated using the secondary Equations (2) and (3) [23].

(2) $$\mathrm{Slope}=\frac{K_{m}}{V_{\max}^{}}+\frac{K_{m}[I]}{V_{\max}^{}K_{i}^{}}$$

(3) $$Y-\mathrm{intercept}=\frac{1}{V_{\max}^{app}}=\frac{1}{V_{\max}^{}}+\frac{1}{\alpha V_{\max}^{}K_{i}^{}}[I]\;$$

#### 2.2.3. Multivariate Curve Resolution–Alternate Least Squares (MCR–ALS) Algorithm

MCR–ALS is acknowledged as a robust chemometric methodology to analyze massive and sophisticated data [24], and was used to obtain the concentration curves and spectral information about hesperetin, PPO and the PPO–hesperetin complex. Fluorescence titration experiment A: The concentration of PPO was 4.0 μM and the same volume of hesperetin was added into PPO solution 25 times (0→20.0 μM). Fluorescence titration experiment B: The concentration of hesperetin was 8.0 μM and the same volume of PPO was added into hesperetin solution 25 times (0→5.0 μM). Two obtained data matrices, *D*^PPO^ and *D*^hesperetin^, were constructed as an extended data matrix [*D*^PPO^, *D*^hesperetin^], which was further decomposed using the following equation.

(4) $$\left[\begin{array}{c} D^{\mathrm{PPO}}\\ D^{\mathrm{hespertin}} \end{array}\right]=\left[\begin{array}{c} C^{\mathrm{PPO}}\\ C^{\mathrm{hespertin}} \end{array}\right]\times S^{T}+\left[\begin{array}{c} E^{\mathrm{PPO}}\\ E^{\mathrm{hespertin}} \end{array}\right]$$

where [*C*^PPO^ *C*^hesperetin^], *S^T^* and [*E*^PPO^ *E*^hesperetin^] are the matrix for pure component concentration, pure spectra and error, respectively. MCR–ALS analysis steps were described in detail in a previous report [21].

#### 2.2.4. Fluorometric Titration Experiments

Fluorescence spectra were measured at 298, 304 and 310 K by a fluorophotometer (F–7000, Hitachi, Tokyo, Japan). Briefly, the concentration of PPO was 2.0 μM, and the same volume of hesperetin was added into the PPO solution (2.0 mL) 10 times (0→25.0 μM). After each addition of hesperetin, 3 min was timed to equilibrate the mixed system. Then, the emission spectra (290–500 nm) were scanned at fixed excitation wavelength (280 nm) [25].

To deduct the effect of internal filtration, all measured fluorescence data were processed through Formula (5) [26]:

(5) $$F_{C}=F_{M}e^{\frac{\left(A_{1}+A_{2}\right)}{2}}$$

where *F*_C_ and *F*_M_ represent the corrected and detected fluorescence, respectively. *A*_1_ and *A*_2_ stand for the absorbance values of hesperetin at excitation and emission wavelengths, respectively.

#### 2.2.5. Synchronous Fluorescence Experiments

Δλ, the difference in wavelength between excitation and emission, was set at 15 and 60 nm to investigate the change of peak. The synchronous fluorescence spectra of PPO solution with the addition of hesperetin (0→25.0 μM) were deducted from 240 to 340 nm [27]. The ratios of synchronous fluorescence quenching (RSFQ = 1 − *F*/*F*_0_) were calculated to evaluate the effect of tryptophan (Trp) and tyrosine (Tyr) residues on quenching the fluorescence of PPO [28].

#### 2.2.6. Three-Dimensional (3D) Fluorescence Experiments

The emission spectra of PPO solution with or without hesperetin were recorded once (200–400 nm) when the excitation wavelength changed every 5 nm (200–400 nm). The concentration of PPO and hesperetin in the 2 mL system were 2.0 μM and 15.0 μM, respectively. The obtained data were analyzed and plotted with Matlab software [29].

#### 2.2.7. CD Spectra

A MOS 450 CD spectrometer (Bio–Logic, Claix, France) was prepared. The CD spectra of samples with various molar ratios (*c*^hesperetin^:*c*^PPO^ = 0:1, 1:1 and 4:1) were recorded from 200 nm to 250 nm. The nitrogen flow remained constant. All scanned spectra were background (0.05 mM PBS, pH 6.8) subtracted [30].

#### 2.2.8. Molecular Docking

The crystal structure of PPO (PDB ID: 2Y9X) and the 3D molecular structure of hesperetin were downloaded from the RCSB Protein Data Bank and PubChem database, respectively. After the energy minimization of hesperetin and the pretreatment of PPO by removing water, adding hydrogen and adding polarity, the docking of hesperetin and PPO was simulated using Discovery Studio 2016 software (BIOVIA, Beijing, China). Both the top hits and the random conformations were set as 100 times. The best binding conformation owned the lowest interaction energy and was analyzed in details [31].

#### 2.2.9. Molecular Dynamics (MD) Simulation

The more comprehensive information on PPO binding with hesperetin was obtained by MD simulations using GROMACS 5.1 software [32]. Similar to molecular docking, PPO removed water molecules. The topology file of hesperetin went through the pretreatment of the Acpype Server before use. Then, the obtained hesperetin combined with PPO to form the total topology file. The AMBER99SB force field was prepared for hesperetin and PPO. The complex was submerged into a regular dodecahedron water box with a 1 nm edge distance. Na^+^ and Cl^−^ were used for charge neutralization. After the procedures of energy minimization, NVT (300 K), and NPT (1 bar) equilibration, 150 ns MD simulation started to run [33].

### 2.3. Statistical Analysis

Results were expressed as means ± standard deviation. The significant differences (*p* < 0.05) were considered through one-way ANOVA, followed by Duncan’s test using IBM SPSS Statistics 26.

## 3. Results

### 3.1. Inhibitory Activity of Hesperetin

**Figure 1:**
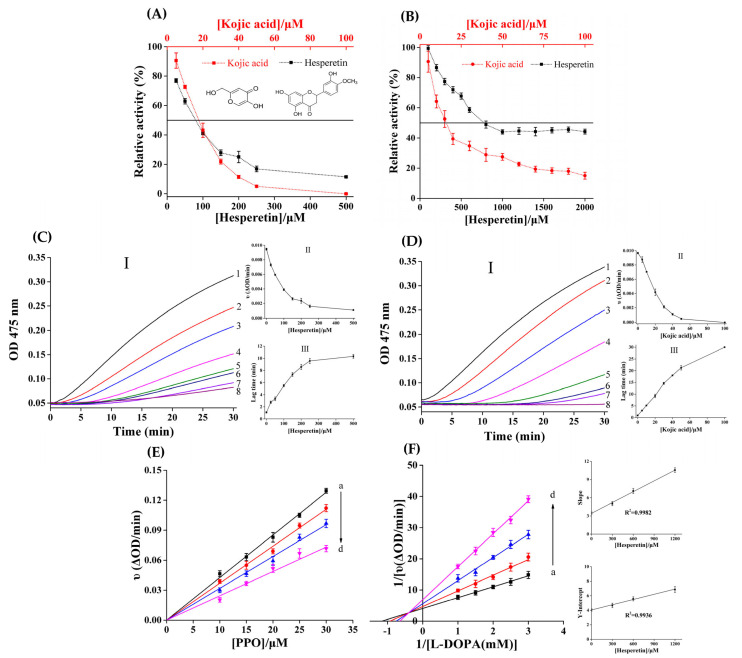
The monophenolase (**A**) and diphenolase (**B**) activity of hesperetin and kojic acid on inhibiting PPO. The inhibition of hesperetin (**C**) and kojic acid (**D**) on monophenolase: (**C-I**,**D-I**) Progress curves for L-tyrosine oxidation by PPO. Curve 1→8: c(hesperetin) = 0, 25, 50, 100, 150, 200, 250, 500 μM and c(kojic acid) = 0, 5, 10, 20, 30, 40, 50, 100 μM. Effect of [hesperetin/kojic acid] on the reaction rate (**C-II**,**D-II**) and the lag time (**C-III**,**D-III**); (**E**) plots of *v* versus [PPO]. c(hesperetin) = 0, 300, 600, 1200 μM for curve a→d, respectively. c(L-dopa) = 0.5 mM; (**F**) Lineweaver–Burk plots for different concentration of hesperetin (0, 300, 600, 1200 μM for curve a→d, respectively). c(PPO) = 0.2 μM. The secondary plots represent Y-intercept and slope versus [hesperetin], respectively.

To examine the inhibitory ability of hesperetin on monophenolase, L-tyrosine was used as a substrate [10]. As the concentration of hesperetin in the system continuously increased, the corresponding residual activity of PPO was gradually attenuated (Figure 1A). The IC_50_ value of hesperetin was 80.8 ± 1.31 μM, while that of kojic acid was 17.7 ± 0.42 μM, suggesting that the inhibitory ability of hesperetin was weaker than the positive control kojic acid. When the concentration was 100.0 μM, hesperetin decreased the residual activity of PPO to 41.2%, while kojic acid almost completely inhibited the catalytic activity of PPO. Figure 1C,D depict the process curves for L-tyrosine oxidation influenced by different concentrations of hesperetin and kojic acid, respectively. Both hesperetin and kojic acid for reaction rate exhibited a dropping trend with the decreasing concentration (Figure 1(C-II,1D-II)). As shown in Figure 1(C-III), when hesperetin was at 500.0 μM, the lag time was extended (1.08→10.33 min). Kojic acid at 100.0 μM lengthened the lag time by more than 30 min (Figure 1(D-III)). A previous study reported that the IC_50_ value of quercetin on the inhibition of monophenolase activity was 19.4 ± 0.35 μM [10]. Comparatively, hesperetin showed lower inhibitory ability on monophenolase. The structural differences in the compounds may be the important reason for their different inhibitory activities [11]. However, hesperetin was still a valid PPO inhibitor, which may prolong the lag time of PPO oxidation and reduce the reaction rate of the enzyme, consequently inhibiting PPO activity [34].

Regarding previous research [23], L-dopa was prepared as a substrate to determine the inhibitory ability of hesperetin on diphenolase. The inhibition of both hesperetin and kojic acid on PPO increased with their concentrations (Figure 1B). Once the concentration of hesperetin was greater than 1.0 mM, the residual activity of PPO no longer continued to decline. Similarly, the inhibitory ability of hesperetin on PPO was weaker than kojic acid, whose IC_50_ values were 776.0 ± 15.53 μM and 16.2 ± 0.37 μM, respectively. The inhibition of hesperetin on PPO was also weaker than previously studied flavonoids, such as norartocarpetin and luteolin [3]. This may be because hesperetin has less hydroxyl and owns methoxy on its C4′ of ring B, with hydrogenation on C2 = C3 [11]. When inhibiting xanthine oxidase’s activity, flavonoids also showed a similar regular pattern. Kaempferol had a higher inhibition rate than kaempferide, while their C4′ of ring B was converted from hydroxyl to methoxy and the hydrogenation of the C2 = C3 weakened the inhibitory ability of flavonoids; that is, quercetin was superior to taxifolin [28]. Thus, the determination of the inhibitory capacity for hesperetin was in line with previous studies, and further provided theoretical support for developing novel PPO inhibitors.

### 3.2. Inhibition Kinetics of Hesperetin

At different concentrations of hesperetin, the effect on the enzymatic reaction rate (ΔOD/min) by increasing the concentrations of PPO were shown in Figure 1E. The four lines intersected at the origin of the coordinate and showed good linearity. As the concentration of hesperetin increased, the slopes of the lines declined. These results implied that hesperetin reversibly inhibited the activity of PPO, and it did not reduce the amount of PPO available but only attenuated its catalytic rate for L-dopa oxidation [35].

The inhibitory effect of hesperetin against PPO is shown in Figure 1F. Four lines intersected in the second quadrant. Either the slopes or the Y-intercept of four lines augmented with the rising concentrations of hesperetin, which represented the values of *K*_m_ increased in a concentration-dependent manner while that of *V*_max_ decreased. These results reflected that hesperetin was the mixed-type inhibitor of PPO [36]. However, a previous study reported that hesperetin was a competitive PPO inhibitor [17]. The inhibition mechanism of hesperetin against PPO deserved further exploration. In addition, the graphs of both Y-intercept and slope versus hesperetin had a good linear relationships, implying that when hesperetin inhibited PPO, there was only one or a class site. According to Equations (2) and (3), the *K*_i_ and *α* values were 569.4 ± 6.24 μM and 2.93, respectively. Thus, hesperetin may have a better chance to bind with free PPO instead of PPO–hesperetin complex [37].

### 3.3. Fluorescence Spectra of PPO–Hesperetin Interaction

**Figure 2:**
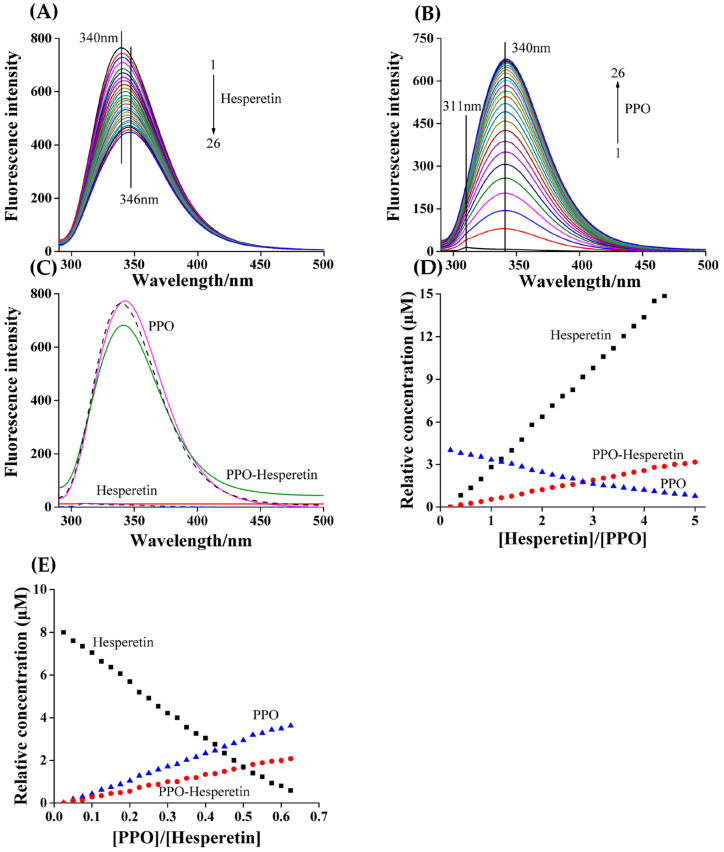
Fluorescence spectra of interaction between PPO and hesperetin. (**A**) Experiment A: c(PPO) = 4.0 μM, and c(hesperetin) = 0, 0.8, 1.6, 2.4, …, 20.0 μM for curves 1→26, respectively. (**B**) Experiment B: c(hesperetin) = 8.0 μM, and c(PPO) = 0, 0.2, 0.4, 0.6, …, 5.0 μM for curves 1→26, respectively. (**C**) MCR–ALS analysis results. Dashed line: measured spectra; solid line: resolved spectra. (**D**) Concentration profiles for experiment A: *C*^PPO^. (**E**) Concentration profiles for experiment B: *C*^hesperetin^.

PPO showed a characteristic fluorescence emission peak at 340 nm. With the gradual addition of hesperetin, the peak red-shifted to 346 nm (Figure 2A). In contrast, the fluorescence emission peak intensity of hesperetin was weak and it was located at 311 nm. With the continuous addition of PPO, a new fluorescence peak appeared at 340 nm and progressively increased in intensity (Figure 2B). The position of peak change may be that PPO interacted with hesperetin, and formed a PPO–hesperetin complex. The extended spectral data matrix [*D*^PPO^, *D*^hesperetin^] was further analyzed using the MCR–ALS algorithm to judge whether the PPO–hesperetin complex was formed or not [24].

### 3.4. MCR–ALS Decomposition

The decomposition of [*D*^PPO^, *D*^hesperetin^] was performed using the singular value decomposition (SVD) model to determine the number of factors associated with chemical species. The first four eigenvalues were 112.75, 42.78, 14.56 and 1.34, implying that three independent factors existed in this complex solution: PPO, hesperetin and PPO–hesperetin complex [24]. As shown in Figure 2C, the resolved profiles of PPO and hesperetin acquired from MCR–ALS analysis (solid lines) overlapped well with the measured profiles (dashed lines), suggesting that the MCR–ALS model correctly resolved the concentration profiles of the components. Either continuously adding hesperetin into the PPO solution (Figure 2D) or continuously adding PPO into the hesperetin solution (Figure 2E), the added hesperetin did not react completely with PPO (and vice versa), so that free hesperetin or PPO existed in the system; the concentration profile of the PPO–hesperetin complex always showed an increasing trend. These results provided powerful evidence that PPO interacted with hesperetin and formed PPO–hesperetin complexes [21].

### 3.5. Intrinsic Fluorescence of PPO Quenched by Hesperetin

**Figure 3:**
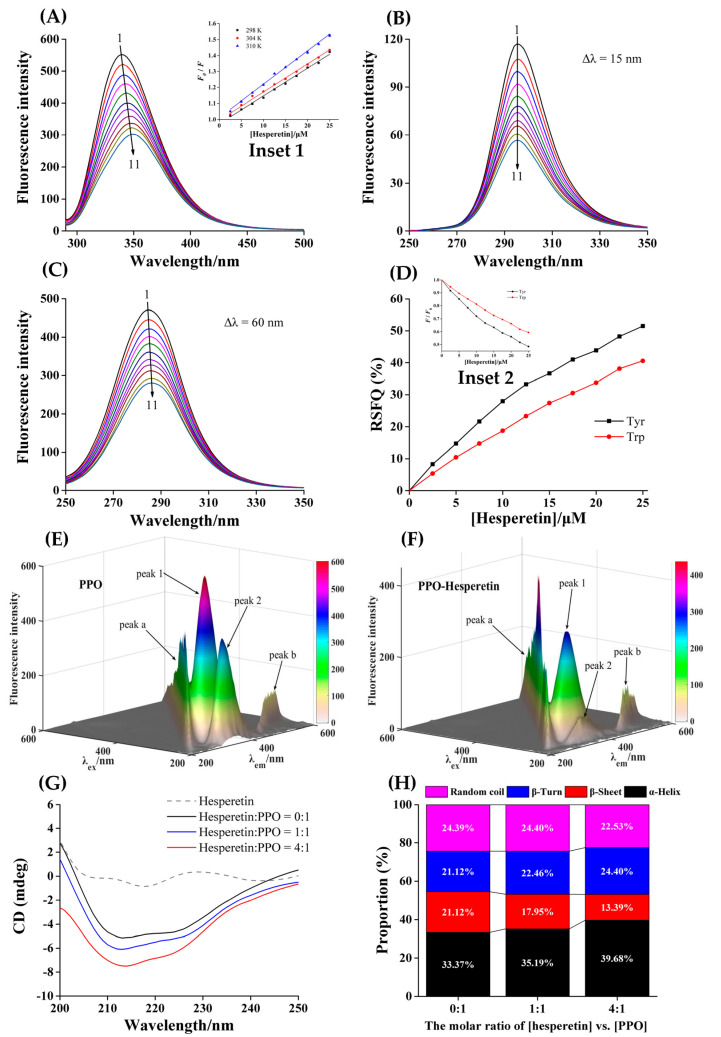
Fluorescence spectra (**A**), synchronous fluorescence spectra Δλ = 15 nm (**B**) and Δλ = 60 nm (**C**) of PPO quenched by hesperetin. c(PPO) = 2.0 μM. Curves 1→11: c(hesperetin) = 0, 2.5, 5.0, 7.5, 10.0, 12.5, 15.0, 17.5, 20.0, 22.5 and 25.0 μM. Insert 1: Stern–Volmer curves at 298, 304 and 310 K. (**D**) RSFQ values of Tyr and Trp. The insert 2 represents plots of *F*/*F*_0_ versus [hesperetin]. Three-dimensional fluorescence spectra of PPO (**E**) and hesperetin–PPO (**F**) systems. c(PPO) = 2.0 μM. c(hesperetin) = 15.0 μM. CD spectra (**G**) and secondary structure content (%) (**H**). c(PPO) = 2.0 μM, the molar ratios of [hesperetin] versus [PPO] were 0:1, 1:1, 4:1, respectively.

The binding between PPO and hesperetin could be reflected by the change of fluorescence spectra. PPO had aromatic amino acids (Trp, Tyr, etc.) so that there was a characteristic fluorescence emission peak at 340 nm. Under the same conditions, hesperetin alone only had a slight peak. The fluorescence intensity of PPO showed a regular downward trend with the gradual addition of hesperetin, suggesting that the interaction occurred between hesperetin and PPO. Furthermore, the position of PPO’s maximum emission peak conspicuously shifted (340→349 nm), indicating that hesperetin may affect the microenvironment of luminescent amino acid residues in PPO (Figure 3A) [38].

### 3.6. Mechanism of Fluorescence Quenching

The Stern–Volmer curves for PPO fluorescence quenched by hesperetin at each temperature were obtained through Equation (6) [39]:

(6) $$\frac{F_{0}}{F}=1+K_{q}\tau_{0}[Q]=1+K_{sv}[Q]$$

where *F* and *F*_0_ denote the fluorescence intensity of PPO with and without hesperetin, respectively. *K_sv_*, *K_q_* and [*Q*] represent the fluorescence quenching constant, quenching rate constant and the concentration of hesperetin, respectively. τ_0_ represents the average fluorophore lifetime without hesperetin, and its value is usually equal to 10^−8^ s [40].

When the temperatures were 298 K, 304 K and 310 K, the *K_sv_* values were (1.71 ± 0.01) × 10^4^, (1.76 ± 0.02) × 10^4^ and (2.09 ± 0.02) × 10^4^ L mol^−1^, respectively (Table 1). At the measured temperatures, the order of magnitude of *K_sv_* was all 10^4^, meaning that the *K_q_* values were all higher than the maximum diffusion collision quenching constant (2.0 × 10^10^ L mol^−1^ s^−1^) [10]. This result signified that PPO bound with hesperetin and formed a static complex, and hesperetin statically quenched PPO’s endogenous fluorescence. The *K*_sv_ values were positively correlated with temperatures, signifying that the increase in temperature favored the fluorescence-quenching effect of hesperetin on PPO [23].

### 3.7. Thermodynamic Parameters and Binding Forces

Based on the following equation, the information about the interaction between hesperetin and PPO were obtained, including binding constant (*K_a_*) and binding number (*n*) [41]:

(7) $$\log\frac{F_{0}-F}{F}=n\log K_{a}-n\log\frac{1}{[Q_{t}]-\frac{\left(F_{0}-F)[P_{t}\right]}{F_{0}}}$$

where [*Q_t_*] and [*P_t_*] stand for the concentrations of hesperetin and PPO. At the experimental temperatures, all lines had a good linear relationship. The *K_a_* values were 1.98 × 10^4^, 2.05 × 10^4^ and 2.22 × 10^4^ L mol^−1^, which were all in the order of 10^4^ L mol^−1^, suggesting that when hesperetin bound to PPO, there was a moderate affinity. The higher the temperature, the higher the *K_a_* values, indicating that the stability of PPO–hesperetin complex may be disturbed by rising temperature [23]. The *n* values at 298, 304 and 310 K were all equal to 1, implying that only one site existed when hesperetin interacted with PPO.

The values of Δ*H*°, Δ*S*° and Δ*G*°, which refer to enthalpy change, entropy change and free energy change, respectively, were obtained by the van ’t Hoff equations [42]:

(8) $$\log K_{a}=-\frac{\Delta H{}^{\circ}}{2.303RT}+\frac{\Delta S{}^{\circ}}{2.303R}$$

(9) ΔG°=ΔH°−TΔS°

where *T* represents temperature; *R* represents gas constant, and its value is 8.314 J mol^−1^ K^−1^.

The negative Δ*G*° values indicated that the combining action of hesperetin and PPO was spontaneous [21]. The positive Δ*H*° value (17.47 KJ mol^−1^) meant that the binding of hesperetin and PPO was an endothermic process. The Δ*S*° value (107.22 J mol^−1^ K^−1^) was also positive (Table 1), indicating that the interaction between hesperetin and PPO made the PPO–hesperetin system become more disordered. A previous study in which hesperetin interacted with glutenin reported the same results [43]. Furthermore, based on the well-known theory of Ross and Subramanian [44], the positive Δ*H*° and Δ*S*° values show that hesperetin was combined with PPO mainly by hydrophobic interaction [33].

### 3.8. Conformational Changes of PPO

The synchronous fluorescence spectra were available for analyzing how hesperetin affected the microenvironment of Tyr and Trp residues in PPO (Figure 3B,C). Their fluorescence intensities were consistently attenuated by the gradual addition of hesperetin (0→25.0 μM). The position of the maximum emission peak for Trp residues had a slight shift (285→287 nm), indicating that hesperetin augmented the polarity of the microenvironment around Trp residues in PPO, while it had no effect on that around Tyr residues. Furthermore, Tyr residues had higher values of RSFQ compared with Trp residues (Figure 3D), implying that Tyr residues may be closer to their binding site, and contribute more when hesperetin quenched PPO’s fluorescence [45].

Another method to judge whether hesperetin changed the conformation of PPO was to compare the 3D fluorescence spectra of PPO and PPO–hesperetin (Figure 3E,F). Peak a (λ_ex_ = λ_em_) and Peak b (2 λ_ex_ = λ_em_) denote the Rayleigh scattering peak and second-order scattering peaks, respectively. Peak 1, the feature of Trp and Tyr residues, whose intensity was weakened (601.2→296.9) and position had a movement (λ_ex_/λ_em_ = 280/340 nm→275/350 nm). The position of peak 2 (the characteristic peak of polypeptide backbone structure generated through π→π* transition) shifted from 230/335 nm to 235/340 nm, and its intensity dropped from 383.8 to 65.9. The alteration of peak 1 further implied that hesperetin influenced Trp and Tyr residues. The change of Peak 2 meant that the addition of hesperetin may change the conformation of PPO as well [46].

The CD spectrum is so sensitive to the change of secondary structure of the protein that is a powerful means to further detect the influence of hesperetin on PPO’s conformation. As shown in Figure 3G, the two negative absorption bands at 208 nm and 222 nm were considered as the characteristic CD spectrum of α-helix structure [47], whose content was positively correlated with its intensity. Thus, hesperetin increased the content of α-helix in PPO [30]. As shown in Figure 3H, with the increase in [hesperetin] versus [PPO] (0:1→1:1→4:1), the contents of α-helix (33.37%→35.19%→39.68%) and β-turn (21.12%→22.46%→24.40%) increased, while the content of β-sheet reduced (21.12%→17.95%→13.39%). This phenomenon meant that hesperetin might induce the structure of PPO to partially contract, and the tighter conformation of the enzyme may make the entry of substrates into the active center more difficult, thus inhibiting PPO activity [18]. In addition to chelating the copper ions in the active center to inhibit PPO activity [17], hesperetin changed PPO’s conformation, which may also be one of the reasons for the inhibition of hesperetin against PPO.

### 3.9. Molecular Docking

**Figure 4:**
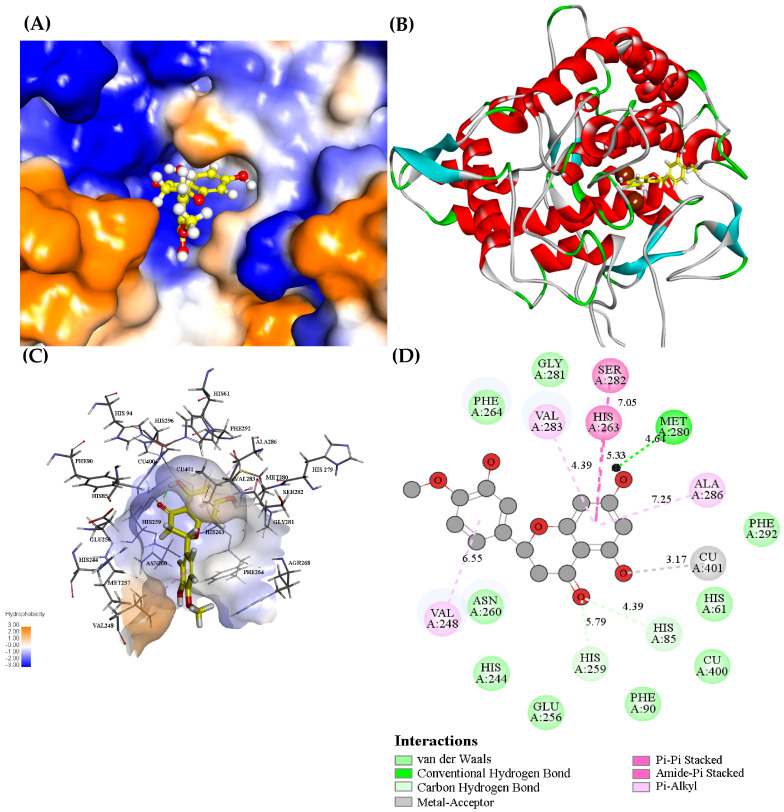
Pattern of binding between hesperetin and PPO: (**A**) Hesperetin binds to the hydrophobic cavity of PPO. Blue and orange represent the hydrophilic and hydrophobic parts of the enzyme surface, respectively. (**B**) The 3D view of binding of hesperetin with PPO. (**C**) Hydrophobic surface and surrounding amino acid residues in the binding area of hesperetin to PPO. (**D**) Amino acid residues of PPO interacting with hesperetin (2D schematic interaction diagram).

The information about the binding site, binding mode and force type for the interaction between hesperetin and PPO could be intuitively observed by molecular docking [48]. Hesperetin entered the cavity of PPO and interacted with surrounding hydrophobic amino acid residues (Figure 4A). Hesperetin bound near the active center (containing two Cu^2+^) of PPO (Figure 4B). The A7–OH of hesperetin formed a hydrogen bond with Met280, whose distance was 4.64 Å. The other two hydrogen bonds were formed between the carbonyl in the C ring of hesperetin and His89 and His259 and the distances were 4.39 Å and 5.79 Å, respectively (Figure 4C,D). Moreover, hesperetin interacted with Val283, Phe264, His85, Asn260, Val248 and His263 via hydrophobic forces, while with Phe292, His61, Phe90, Glu256, His244, Asn260, Phe264 and Gly281 via van der Waals forces. Thus, hydrogen bonds, hydrophobic interactions and van der Waals forces were all important forces in the formation of the PPO–hesperetin complex.

Si et al. [17] also simulated the molecular docking of hesperetin with PPO using two different docking programs. They found that Met280, His61, His85 and His259 in PPO were important sites in forming hydrogen bonds with hesperetin. The findings in our study were similar to the report. It is noteworthy that hesperetin interacted with two copper ions and the critical residues around the active center of PPO. This phenomenon was also found by Song et al. [23], who reported that the interactions between the compound and PPO may influence the stabilization of the dinuclear copper active center of PPO.

All in all, hesperetin entered PPO’s hydrophobic cavity, bound near its active center, acted as an obstruction to occupy the substrate’s binding site in PPO, prevented the substrate being catalyzed by PPO and may change the structure of PPO as well, thus leading to decreased PPO activity [49].

### 3.10. Molecular Dynamics

Experiments can only give macroscopic results, while MD simulations can provide atomic-level insights into the binding of hesperetin to PPO. Therefore, this method can be used to supplement and verify the known results.

**Figure 5:**
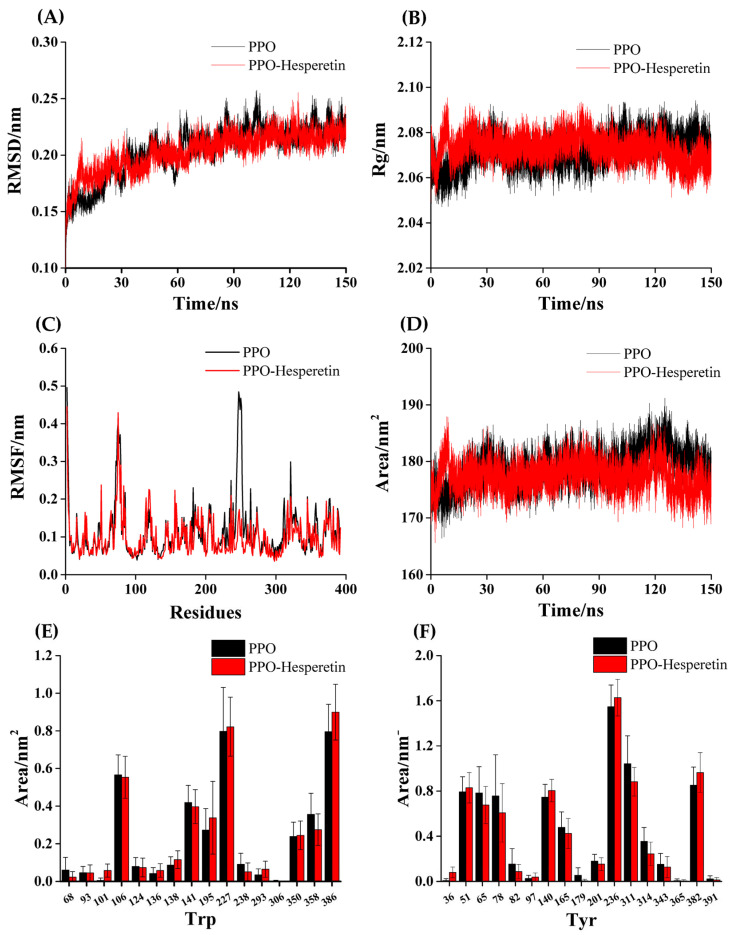
MD simulation of hesperetin with PPO for 150 ns. The RMSD (**A**), Rg (**B**), RMSF (**C**) and SASA (**D**) plots of PPO and PPO–hesperetin complexes. The SASA values of Trp (**E**) and Tyr (**F**).

The root mean square deviation (RMSD) value represents the stability of the systems [23]. By comparing free PPO with PPO–hesperetin complex in RMSD values, we could determine whether hesperetin affects the stability of PPO. The more stable systems have less variation in RMSD values. The RMSD values of PPO and PPO–hesperetin systems tended to increase, reached a relatively stable state after 100 ns, and finally stabilized at around 0.22 nm (Figure 5A). The RMSD values of PPO–hesperetin had less volatility in the first 30 ns, which may be caused by the formation of hydrogen bonds in the complex system [50]. After stabilization, the complex owned the lower RMSD values, implying that adding hesperetin into the system improved its stability [51].

The radius of gyration (Rg) value is negatively correlated with the structural denseness of the enzyme [52]. The Rg value of the PPO–hesperetin system remained at around 2.065 nm, which was smaller than the free PPO system at 2.075 nm (Figure 5B), suggesting that hesperetin may strengthen the tightness of the PPO structure [42].

The root mean square fluctuation (RMSF) value reflects the flexibility of amino acid residues in PPO influenced by hesperetin [53]. Overall, the free PPO system had a higher RMSF value than the complex system; in particular, the residues 240–260 (Figure 5C). This result suggested that hesperetin could improve the stability of the PPO structure by restricting some amino acids, which may be involved in the binding process of hesperetin with PPO.

The solvent-accessible surface area (SASA) value reflects the change in the surface area between the system and the solvent, and its value increases with the decreasing hydrophobicity of the enzyme [23]. The decreased SASA value (Figure 5D) indicated that hesperetin reduced PPO’s hydrophilicity and tightened its structure [54]. Hesperetin only had a feeble effect on the microenvironment of Trp and Tyr in PPO (Figure 5E,F). All the MD simulation results were consistent with the results from fluorescence and CD spectra experiments.

## 4. Conclusions

Hesperetin could inhibit the oxidation of L-tyrosine and L-dopa catalyzed by PPO, which was a reversible and mixed-type PPO inhibitor. MCR–ALS analysis showed that PPO interacted with hesperetin and formed the PPO–hesperetin complex. The combination of hesperetin and PPO was mainly driven by hydrophobic interactions, while only one site existed for their binding. The addition of hesperetin affected the microenvironment of residues in PPO and increased PPO’s α-helix content, which represented the tighter molecular structure of PPO, and thus may make the entry of the substrate into, and the release of the catalytic products from, the active center more complex, leading to decreased PPO activity. This study may provide novel insights into the inhibition mechanism of hesperetin against PPO and theoretical guidance for the development of new PPO inhibitors. However, the anti-browning effect of hesperetin in real food systems need be further investigated.

## Figures and Tables

**Table 1:** Quenching constants *K*_sv_, binding constants *K*_a_, number of binding sites *n* and thermodynamic parameters.

*T* (K)	*K*_*sv*_ (×10^4^ L mol^−1^)	*R* ^a^	*K*_*a*_ (×10^4^ L mol^−1^)	*R* ^b^	*n*	Δ*H*° (kJ mol^−1^)	Δ*G*° (kJ mol^−1^)	Δ*S*° (J mol^−1^K^−1^)
298	1.71 ± 0.01 ^a^	0.9982	1.98 ± 0.01 ^a^	0.9988	1.23 ± 0.03	17.47	−24.49	107.22
304	1.76 ± 0.02 ^b^	0.9976	2.05 ± 0.02 ^b^	0.9914	1.08 ± 0.01		−25.13	
310	2.09 ± 0.02 ^c^	0.9979	2.22 ± 0.05 ^c^	0.9987	0.99 ± 0.04		−25.77	

*R*^a^ is the correlation coefficient for the *K_sv_* values; *R*^b^ is the correlation coefficient for the *K_a_* values. Different letters represent significant differences (*p* < 0.05).

## Data Availability

The data are available from the corresponding author.

## Abbreviations

PPO	polyphenol oxidase
IC_50_	half-maximum inhibitory concentration
MCR–ALS	multivariate curve resolution–alternate least squares
CD	circular dichroism
SVD	singular value decomposition
RSFQ	ratios of synchronous fluorescence quenching
3D	three-dimensional
MD	molecular dynamics
RMSD	root mean square deviation
Rg	radius of gyration
RMSF	root mean square fluctuation
SASA	solvent accessible surface area

## References

[1] Huang G.L., Liu T.T., Ma J.J., Sun X.L., Sui S.Y., Quan X.Y., Wang Y.M. (2022). Anti-polyphenol oxidase mechanism of oligomeric procyanidins and its application on browning control of “Baiyu” loquat during storage. Food Biosci..

[2] Alizadeh N., Sayahi M.H., Iraji A., Yazdaf R., Moazzam A., Mobaraki K., Adib M., Attarroshan M., Larijani B., Rastegar H. (2022). Evaluating the effects of disubstituted 3-hydroxy-1H-pyrrol-2(5H)-one analog as novel tyrosinase inhibitors. Bioorg. Chem..

[3] Zhang L., Zhao X., Tao G.J., Chen J., Zheng Z.P. (2017). Investigating the inhibitory activity and mechanism differences between norartocarpetin and luteolin for tyrosinase: A combinatory kinetic study and computational simulation analysis. Food Chem..

[4] Xiong Z.Q., Zhou L., Liu W. (2015). Research progress in the inhibition of Polyphenol oxidase. Sci. Technol. Food Ind..

[5] Zhang Y., Sun Y., Zhang H., Mai Q., Zhang B., Li H., Deng Z. (2020). The degradation rules of anthocyanins from eggplant peel and antioxidant capacity in fortified model food system during the thermal treatments. Food Biosci..

[6] Zhou H.L., Wang F.H., Yi J.J., Cheng F.Y., Yuan L., Niu H.H., Zhou L.Y. (2021). Research progress on the effect of chemical inhibitors on the properties of polyphenol oxidase in fruits and vegetables. Food Ferment. Ind..

[7] Lee M.K., Hwang Y.H., Ryu H., Lee A., Jeong H.H., Baek J., Kim M.J., Lee J.Y., Van J.Y., Liu Y.S. (2022). Galla rhois water extract inhibits enzymatic browning in apple juice partly by binding to and inactivating polyphenol oxidase. Food Chem..

[8] Siew Z.Z., Chan E.W.C., Wong C.W. (2022). Hydrophobic bioactive constituents of cinnamon bark as inhibitor of polyphenol oxidase from Musa acuminata ‘Mas’ peel. Biocatal. Agric. Biotechnol..

[9] Yu K.B., Zhou L., Sun Y.F., Zeng Z.C., Chen H.W., Liu J.P., Zou L.Q., Liu W. (2021). Anti-browning effect of Rosa roxburghii on apple juice and identification of polyphenol oxidase inhibitors. Food Chem..

[10] Fan M.H., Zhang G.W., Hu X., Xu X.M., Gong D.M. (2017). Quercetin as a tyrosinase inhibitor: Inhibitory activity, conformational change and mechanism. Food Res. Int..

[11] Fan M.H., Ding H.F., Zhang G.W., Hu X., Gong D.M. (2019). Relationships of dietary flavonoid structure with its tyrosinase inhibitory activity and affinity. LWT.

[12] Xiong Z.Q., Liu W., Zhou L., Zou L.Q., Chen J. (2016). Mushroom (Agaricus bisporus) polyphenoloxidase inhibited by apigenin: Multi-spectroscopic analyses and computational docking simulation. Food Chem..

[13] Jiang H.W., Zhou L., Sun Y.F., Yu K.B., Yu W.Z., Tian Y.Q., Liu J.P., Zou L.Q., Liu W. (2022). Polyphenol oxidase inhibited by 4-hydroxycinnamic acid and naringenin: Multi-spectroscopic analyses and molecular docking simulation at different pH. Food Chem..

[14] Parhiz H., Roohbakhsh A., Soltani F., Rezaee R., Iranshahi M. (2015). Antioxidant and anti-inflammatory properties of the citrus flavonoids hesperidin and hesperetin: An updated review of their molecular mechanisms and experimental models. Phytother. Res..

[15] Roohbakhsh A., Parhiz H., Soltani F., Rezaee R., Iranshahi M. (2015). Molecular mechanisms behind the biological effects of hesperidin and hesperetin for the prevention of cancer and cardiovascular diseases. Life Sci..

[16] Moghaddam A.H., Zare M. (2018). Neuroprotective effect of hesperetin and nano-hesperetin on recognition memory impairment and the elevated oxygen stress in rat model of Alzheimer’s disease. Biomed. Pharmacother..

[17] Si Y.X., Wang Z.J., Park D., Chung H.Y., Wang S.F., Yan L., Yang J.M., Qian G.Y., Yin S.J., Park Y.D. (2012). Effect of hesperetin on tyrosinase: Inhibition kinetics integrated computational simulation study. Int. J. Biol. Macromol..

[18] Xu H., Li X., Mo L., Zou Y., Zhao G. (2022). Tyrosinase inhibitory mechanism and the anti-browning properties of piceid and its ester. Food Chem..

[19] Li G.H., Lee Y.Y., Lu X.X., Chen J., Liu N., Qiu C.Y., Wang Y. (2022). Simultaneous loading of (−)-epigallocatechin gallate and ferulic acid in chitosan-based nanoparticles as effective antioxidant and potential skin-whitening agents. Int. J. Biol. Macromol..

[20] Karakaya G., Türe A., Ercan A., Öncül S., Aytemir M.D. (2019). Synthesis, computational molecular docking analysis and effectiveness on tyrosinase inhibition of kojic acid derivatives. Bioorg. Chem..

[21] Ding H.F., Wu X.Q., Pan J.H., Hu X., Gong D.M., Zhang G.W. (2018). New insights into the inhibition mechanism of betulinic acid on α-glucosidase. J. Agric. Food Chem..

[22] Li J., Gong Y.H., Li J.W., Fan L.P. (2022). In vitro inhibitory effects of polyphenols from Tartary buckwheat on xanthine oxidase: Identification, inhibitory activity, and action mechanism. Food Chem..

[23] Song X., Ni M.T., Zhang Y., Zhang G.W., Pan J.H., Gong D.M. (2021). Comparing the inhibitory abilities of epigallocatechin-3-gallate and gallocatechin gallate against tyrosinase and their combined effects with kojic acid. Food Chem..

[24] Li S., Hu X., Pan J.H., Gong D.M., Zhang G.W. (2021). Mechanistic insights into the inhibition of pancreatic lipase by apigenin: Inhibitory interaction, conformational change and molecular docking studies. J. Mol. Liq..

[25] Pu P., Zheng X., Jiao L.N., Chen L., Yang H., Zhang Y.H., Liang G.Z. (2021). Six flavonoids inhibit the antigenicity of β-lactoglobulin by noncovalent interactions: A spectroscopic and molecular docking study. Food Chem..

[26] Maurya N., Maurya J.K., Singh U.K., Dohare R., Zafaryab M., Moshahid Alam Rizvi M., Kumari M., Patel R. (2019). In vitro cytotoxicity and interaction of noscapine with human serum albumin: Effect on structure and esterase activity of HSA. Mol. Pharm..

[27] Cheng D., Wang X.R., Tang J.L., Zhang X.Y., Wang C.L., Li H. (2019). Characterization of the binding mechanism and conformational changes of bovine serum albumin upon interaction with aluminum-maltol: A spectroscopic and molecular docking study. Metallomics.

[28] Zhao J., Huang L., Sun C.Y., Zhao D.S., Tang H.J. (2020). Studies on the structure-activity relationship and interaction mechanism of flavonoids and xanthine oxidase through enzyme kinetics, spectroscopy methods and molecular simulations. Food Chem..

[29] Huang G., Jin H.N., Liu G.C., Yang S.Y., Jiang L.Z., Zhang Y., Sui X.N. (2022). An insight into the changes in conformation and emulsifying properties of soy β-conglycinin and glycinin as affected by EGCG: Multi-spectral analysis. Food Chem..

[30] Murtaza A., Iqbal A., Linhu Z., Liu Y., Xu X., Pan S., Hu W. (2019). Effect of high-pressure carbon dioxide on the aggregation and conformational changes of polyphenol oxidase from apple (Malus domestica) juice. Innov. Food Sci. Emerg. Technol..

[31] Singh M., Thrimawithana T., Shukla R., Adhikari B. (2022). Inhibition of enzymes associated with obesity by the polyphenol-rich extracts of Hibiscus sabdariffa. Food Biosci..

[32] Wang C., Chen L., Lu Y.C., Liu J., Zhao R., Sun Y.H., Sun B.Y., Wang C.N. (2021). pH-Dependent complexation between β-lactoglobulin and lycopene: Multi-spectroscopy, molecular docking and dynamic simulation study. Food Chem..

[33] Liu C., Lv N., Ren G., Wu R., Wang B., Cao Z., Xie H. (2021). Explore the interaction mechanism between zein and EGCG using multi-spectroscopy and molecular dynamics simulation methods. Food Hydrocoll..

[34] Liu L.L., Li J.D., Zhang L.L., Wei S.D., Qin Z.Y., Liang D.D., Ding B.M., Chen H., Song W. (2022). Conformational changes of tyrosinase caused by pentagalloylglucose binding: Implications for inhibitory effect and underlying mechanism. Food Res. Int..

[35] Hu C.M., Zheng Y.Y., Lin A.T., Zhang X., Wu X.Z., Lin J., Xu X.T., Xiong Z. (2023). Design, synthesis and evaluation of indole-based bisacylhydrazone derivatives as α-glucosidase inhibitors. J. Mol. Struct..

[36] Mahdavi A., Mohammadsadeghi N., Mohammadi F., Saadati F., Nikfard S. (2022). Evaluation of inhibitory effects of some novel phenolic derivatives on the mushroom tyrosinase activity: Insights from spectroscopic analyses, molecular docking and in vitro assays. Food Chem..

[37] Zheng L., Lee J., Yue L.M., Lim G.T., Yang J.M., Ye Z.M., Park Y.D. (2018). Inhibitory effect of pyrogallol on α-glucosidase: Integrating docking simulations with inhibition kinetics. Int. J. Biol. Macromol..

[38] Santa Rosa L.N., de Paula Rezende J., Coelho Y.L., Mendes T.A.O., da Silva L.H.M., dos Santos Pires A.C. (2021). β-lactoglobulin conformation influences its interaction with caffeine. Food Biosci..

[39] Wen Y.X., Zhou X., Huo D., Chen J.C., Weng L.M., Li B., Wu Z.Q., Zhang X., Li L. (2022). Optimization for the extraction of polysaccharides from Huidouba and their in vitro α-glucosidase inhibition mechanism. Food Biosci..

[40] Fu J.J., Sun C., Tan Z.F., Zhang G.Y., Chen G.B., Song L. (2022). Nanocomplexes of curcumin and glycated bovine serum albumin: The formation mechanism and effect of glycation on their physicochemical properties. Food Chem..

[41] Shi R.J., Chen W., Pan F., Zhao P.P., He Y.T., Yu R., Fu R.X., Munkh-Amgalan G., Jiang Z.M. (2022). Characterization of the binding behavior, structure and foaming properties of bovine α-lactalbumin combined with saponin by the multi-spectroscopic and silico approaches. Food Hydrocoll..

[42] Sadeghi-Kaji S., Shareghi B., Saboury A.A., Farhadian S. (2020). Investigating the interaction of porcine pancreatic elastase and propanol: A spectroscopy and molecular simulation study. Int. J. Biol. Macromol..

[43] Jiang H.H., Hu X., Pan J.H., Gong D.M., Zhang G.W. (2022). Effects of interaction between hesperetin/hesperidin and glutenin on the structure and functional properties of glutenin. LWT.

[44] Ross P.D., Subramanian S. (1981). Thermodynamics of protein association reactions: Forces contributing to stability. Biochemistry.

[45] Huang S., Peng S.S., Zhu F.W., Lei X.L., Xiao Q., Su W., Liu Y., Huang C.S., Zhang L.X. (2016). Multispectroscopic investigation of the interaction between two ruthenium (II) arene complexes of curcumin analogs and human serum albumin. Biol. Trace Elem. Res..

[46] Yu Z.Y., Xu K., Wang X., Wen Y.T., Wang L.J., Huang D.Q., Chen X.X., Chai W.M. (2022). Punicalagin as a novel tyrosinase and melanin inhibitor: Inhibitory activity and mechanism. LWT.

[47] Greenfield N.J. (1999). Applications of circular dichroism in protein and peptide analysis. TrAC-Trends Anal. Chem..

[48] Zhou H.L., Wang F.H., Niu H.H., Yuan L., Tian J., Cai S.B., Bi X.F., Zhou L.Y. (2022). Structural studies and molecular dynamic simulations of polyphenol oxidase treated by high pressure processing. Food Chem..

[49] Feng Y., Sun Y., Meng Z., Sui X., Zhang D., Yan H., Wang Q. (2022). S-Ethyl thioacetate as a natural anti-browning agent can significantly inhibit the browning of fresh-cut potatoes by decreasing polyphenol oxidase activity. Sci. Hortic..

[50] Das S., Hazarika Z., Sarmah S., Baruah K., Rohman M.A., Paul D., Jha A.N., Roy A.S. (2020). Exploring the interaction of bioactive kaempferol with serum albumin, lysozyme and hemoglobin: A biophysical investigation using multi-spectroscopic, docking and molecular dynamics simulation studies. J. Photochem. Photobiol. B Biol..

[51] Kooravand M., Asadpour S., Haddadi H., Farhadian S. (2021). An insight into the interaction between malachite green oxalate with human serum albumin: Molecular dynamic simulation and spectroscopic approaches. J. Hazard. Mater..

[52] Ni M.T., Hu X., Gong D.M., Zhang G.W. (2020). Inhibitory mechanism of vitexin on α-glucosidase and its synergy with acarbose. Food Hydrocoll..

[53] Lu Y., Zhao R., Wang C., Zhang X., Wang C. (2022). Deciphering the non-covalent binding patterns of three whey proteins with rosmarinic acid by multi-spectroscopic, molecular docking and molecular dynamics simulation approaches. Food Hydrocoll..

[54] Zhou H., Bie S., Li J., Yuan L., Zhou L. (2023). Comparison on inhibitory effect and mechanism of inhibitors on sPPO and mPPO purified from ‘Lijiang snow’ peach by combining multispectroscopic analysis, molecular docking and molecular dynamics simulation. Food Chem..

